# What teachers need to know and be able to do: A view from teachers, students, and principals in the Brazilian context

**DOI:** 10.1371/journal.pone.0238990

**Published:** 2020-09-14

**Authors:** Natalia P. Montoya, Lia C. O. B. Glaz, César C. C. Abad, Lucas A. Pereira, Irineu Loturco

**Affiliations:** 1 Península Institute, São Paulo, Brazil; 2 NAR - Nucleus of High Performance in Sport, São Paulo, Brazil; 3 Department of Human Movement Sciences, Federal University of São Paulo, São Paulo, Brazil; 4 University of South Wales, Pontypridd, Wales, United Kingdom; University of Reading, UNITED KINGDOM

## Abstract

The aim of this study was to determine the main characteristics of a “good teacher” through the use of questionnaires designed to assess teaching skills and competences, considering the point of view of teachers, principals, and students. In total, 82 teachers, 14 principals, and 625 middle-school students from 5 public schools in São Paulo state participated in this study. Two questionnaires were applied, one designed for teachers and principals and the other for students. First, teachers and principals completed their specific questionnaire, after which the other questionnaire was applied to the students. Both questionnaires contained multiple choice questions related to eight distinct subsections. The questions were answered through the use of a Likert scale, varying from 1 (“totally disagree”) to 5 (“totally agree”). The comparisons of the frequency of responses among all questionnaire subsections between teachers and principals were analyzed using a Chi-Square and the z-test, with *P*-values adjusted to the Bonferroni method. The statistical significance level was set as *P* < 0.05. The subsection “class atmosphere” presented the highest percentage of response “totally agree”, closely followed by “professional engagement”. Significant differences (*P* < 0.05) in responses were observed between teachers and principals for “teaching planning and practice”, “use of time and material resources to develop classes”, and “professional engagement” domains. In summary, it was demonstrated that some teaching characteristics might be more important than others, with some of these characteristics exhibiting significant differences between groups. Nevertheless, it is crucial to emphasize that all assessed educational domains may be recognized as critical teaching qualities, as all of them presented high levels of “totally agree” responses.

## Introduction

Excellence in education is at the heart of human, social, and economic development, and current research suggests that teaching quality and practices are probably the most important factors influencing student learning outcomes and achievement in the school environment [1–3]. Numerous studies have suggested that successful teachers play a vital role in human capital formation and development [4], and revealed that countries with a high level of human capital are more likely to present faster and steadier economic growth [3, 5, 6]. Undoubtedly, this phenomenon is highly impacted by the effectiveness of learning processes and quality of the educational systems of these respective countries [6, 7].

In this context, in a large number of developing countries, a framework of policies has been developed in recent decades to provide access to basic education for all children as a response to an international commitment to education for all, leading to 91% of children being enrolled in schools, according to the United Nations Development Program (2015) [8]. Although these policies have been important to guarantee children´s right to schooling, in recent decades, the majority of these countries have experienced a learning crisis [9], as demonstrated by their low levels of literacy and math proficiency, verified by specialized assessments [10]. Consequently, although more children have been granted access to schools, they do not demonstrate the expected level of learning and, as a result, human capital development is compromised [11]. As human capital development is intrinsically linked to learning, it is crucial to more comprehensively understand the characteristics able to impact education quality [6, 12].

Among numerous factors, effective teaching is recognized as key for promoting effective learning and academic achievement [3, 4, 13]. In fact, it is widely accepted that high quality teachers are among the most valuable and relevant assets of schools [3]. As a consequence, it is essential to determine the remarkable attributes of successful teachers. As discussed by Hanushek and Woessmann [14], “the inability to identify specific teacher qualities associated with higher student achievements makes it difficult to regulate the presence of high-quality teachers in classrooms”. To meet this requirement, many countries that obtained successful performances in their educational programs have implemented “National Standards for Good Teaching”, in an attempt to provide a more comprehensive vision of teaching practice and methods, develop more efficient teacher education programs, and describe a series of optimal competences for a “good teacher” [15–18]. From an applied standpoint, the adoption of these formal practices may be considered, at the same time, as a great challenge and a necessary step for developing more promising and effective educational policies [19–21].

Currently, in Brazil, this discussion is incipient [22]. The institution of the first National Common Core for Brazilian Students in 2017 opened the floor to discussion on how to better define teaching quality in the country, given the new set of skills required in schools. In 2018, the Federal Ministry of Education launched the first version of a set of teachers’ competencies called *Base Nacional da Formação Docente* [23] and, then, in 2019, a group of States and Municipalities established a formal working group to discuss the institution of a common understanding of what good teaching means within the Brazilian context. This has been strongly inspired by the national Chilean [24], Canadian, and Australian [25] processes; countries that differ greatly in cultural background and economic status, but which have exhibited a steady and clear improvement in the quality of their educational systems and hence, in their human capital [26].

For this purpose, the aforementioned countries utilized valid and practical instruments (e.g., questionnaires and inquiry forms) to precisely measure the quality of their educational systems [27, 28]. As Brazil is a developing country of continental dimensions and with very high levels of social and economic disparities [29], it is important to assess the efficiency of a similar and regionally adapted approach to evaluate the teaching quality in this country. Based on these outcomes, principals and researchers could develop more efficient monitoring tools, providing teachers and students with meaningful resources and pathways to support learning and achievement. In addition, and more importantly, government agencies could use these reference data to create more effective and tailored educational policies, which is of fundamental importance for the development of the country. Therefore, the question to be answered in this research is: what are the main characteristics of a “good teacher” in the Brazilian context? For this purpose, we used questionnaires specifically designed to collect data regarding teaching skills and competences, considering the point of view of teachers, principals (i.e., directors and coordinators), and students.

## Materials and methods

### Study design

In this cross-sectional study we assessed and identified the main characteristics of “good teachers” in the Brazilian educational scenario. To improve the consistency of our findings we opted to select the schools with the highest scores (a score of at least “6” out of a maximum of “10”) on the national educational index (i.e., *Índice de Desenvolvimento da Educação Básica* [IDEB]), since it is expected that good schools achieve good learning outcomes and have skilled and experienced teachers. The outputs collected from two official instruments commonly used in Chile were assessed, in three different groups (i.e., teachers, principals, and students). The research took place in São Paulo, Brazil, from May to August 2019. To mobilize the target groups, initially, the Regional Education Directories were contacted. Subsequently, each Directory contacted the schools in order to organize and program the data collection procedures. In total, in the latest edition of the test, 7 schools met the necessary score on the national educational index, according to the established criteria, and 5 schools volunteered to participate in the research. In all schools, questionnaires were applied at two different moments: firstly, the teachers and principals completed their specific questionnaire and, subsequently, the specific questionnaire for students was applied. At both moments of data collection, the principal led the activities with the support of the research team.

### Participants

All the eligible population within the 5 selected schools, comprising 82 teachers (age range: 33–47), 14 principals (age range: 44–65), and 625 middle-school students (age range: 12–14) volunteered to participate in this study. Prior to the assessment, the participants received instructions on the formal procedures to complete the instrument. The maximum time to respond to the questionnaires was 90 minutes for teachers and principals and 30 minutes for students. The participants and their legal guardians gave written informed consent, as outlined in the PLOS consent form, to publish this study. This study was performed in accordance with the ethical standards of the Helsinki Declaration and was approved by the Anhanguera-Bandeirante University Ethics Committee.

### Assessment tools

Two distinct questionnaires were used in this study (submitted as supporting information); one specifically designed for teachers and principals, and the other for students. The questionnaire designed for teachers contained 122 questions, while the students’ questionnaire contained 31 questions. Both tools were divided into 8 domains/subsections as follows: 1) “teaching planning and practice”; 2) “efficient use of time and material resources during classes”; 3) “strategies and activities to promote/stimulate meaningful learning”; 4) “knowledge”; 5) “strategies and actions for student assessment”; 6) “comprehensive use of assessment results”; 7) “class atmosphere”; and 8) “professional engagement”. Each subsection contained a different number of questions, which were designed on a Likert-type scale ranging from 1 (“totally disagree”) to 5 (“totally agree”). The frequency of each response in the distinct questionnaire domains was retained for data analysis purposes.

### Statistical analysis

The statistical analyses were performed using the SPSS^®^ software package, version 20.0 (SPSS, Inc., Chicago, IL, USA). Descriptive statistics are presented as the frequency of responses for each questionnaire subsection. The comparisons of the frequency of responses among all questionnaire subsections and between teachers and principals for each questionnaire subsection were analyzed using a Chi-Square and the z-test, with *P*-values adjusted to the Bonferroni method. The statistical significance level was set as *P*< 0.05. Cronbach’s coefficient alpha was calculated for internal consistency of each questionnaire. The estimate of reliability, Cronbach’s alpha, was required to be higher than 0.7. As previously mentioned, we did not compare students with principals and teachers. Therefore, it is not possible to draw definitive conclusions regarding the differences in the responses between these groups. In this regard, based on the descriptive statistics, we decided to discuss the differences greater than 10% in the frequency of responses for each questionnaire domain.

## Results

Table 1 depicts the Cronbach’s alpha in the distinct questionnaire subsections for principals, teachers, and students. The Cronbach’s alpha values were all >0.70. Table 2 demonstrates the main scores among principals, teachers, and students for the percentages of responses “totally agree” for each questionnaire subsection. The subsection “class atmosphere” presented the highest percentage of response “totally agree”, closely followed by “professional engagement”, while the subsection “use of time and material resources to develop classes” presented the lowest percentage of response. Table 3 shows the number and percentages of responses of principals and teachers, and students for each questionnaire subsection. No significant differences were observed in the distribution of the frequency of responses among the distinct subsections for both groups analyzed (*P*> 0.05). Table 4 depicts the comparison of the distribution of the frequency of responses of each questionnaire subsection along with *P*-values, between principals and teachers. Significant differences (*P*< 0.05) in the distribution of responses were observed between principals and teachers for the “teaching planning and practice”, “efficient use of time and material resources to develop classes”, and “professional engagement” domains. No significant differences were observed in the distribution of responses between principals and teachers *P*> 0.05 in the other questionnaire subsections.

**Table 1:** Cronbach’s alpha values for principals, teachers, and students in each questionnaire subsection.

Subsections	Cronbach’s Alpha		
	Principals	Teachers	Students
Class atmosphere	0.71	0.75	0.73
Professional engagement	0.70	0.83	0.78
Comprehensive use of assessment results	0.81	0.79	0.76
Strategies/actions for student assessment	0.78	0.89	0.75
Teaching planning and practice	0.72	0.79	0.70
Strategies and activities to promote/stimulate meaningful learning	0.93	0.90	0.82
Knowledge	0.83	0.75	0.79
Efficient use of time and material resources during classes	0.71	0.74	0.72

**Table 2:** Mean scores among principals, teachers, and students of the percentages of responses “totally agree” for each questionnaire subsection.

Subsections	% of responses
Class atmosphere	80.4
Professional engagement	79.3
Comprehensive use of assessment results	77.0
Strategies/actions for student assessment	72.6
Teaching planning and practice	71.0
Strategies and activities to promote/stimulate meaningful learning	68.4
Knowledge	63.4
Efficient use of time and material resources during classes	57.9

**Table 3:** Number and percentages of responses of principals and teachers, and students for each questionnaire subsection.

Subsections		Principals and Teachers		Students	
		n	%	n	%
Teaching planning and practice	Totally disagree	12	0.7	11	0.4
	Partially disagree	27	1.5	17	0.7
	Nor agree, nor disagree	70	4.0	54	2.2
	Partially agree	510	28.9	486	19.6
	Totally agree	1145	64.9	1911	77.1
Efficient use of time and material resources during classes	Totally disagree	11	0.9	31	1.7
	Partially disagree	57	4.4	78	4.2
	Nor agree, nor disagree	83	6.4	55	3.0
	Partially agree	438	33.9	551	29.7
	Totally agree	703	54.4	1139	61.4
Strategies and activities to promote/stimulate meaningful learning	Totally disagree	7	0.3	56	2.3
	Partially disagree	29	1.0	153	6.2
	Nor agree, nor disagree	44	1.6	123	4.9
	Partially agree	607	21.9	629	25.3
	Totally agree	2089	75.3	1527	61.4
Knowledge	Totally disagree	2	0.3	78	3.3
	Partially disagree	20	3.1	134	5.6
	Nor agree, nor disagree	25	3.9	116	4.9
	Partially agree	196	30.6	515	21.6
	Totally agree	398	62.1	1537	64.6
Strategies/actions for student assessment	Totally disagree	9	0.5	9	0.5
	Partially disagree	77	4.4	29	1.6
	Nor agree, nor disagree	60	3.4	27	1.5
	Partially agree	513	29.1	262	14.1
	Totally agree	1101	62.6	1536	82.5
Comprehensive use of assessment results	Totally disagree	4	0.5	10	0.4
	Partially disagree	8	1.1	60	2.4
	Nor agree, nor disagree	19	2.6	70	2.8
	Partially agree	135	18.2	452	18.0
	Totally agree	576	77.6	1914	76.4
Class atmosphere	Totally disagree	3	0.3	22	0.7
	Partially disagree	16	1.3	66	2.1
	Nor agree, nor disagree	13	1.1	74	2.4
	Partially agree	159	13.3	559	18.0
	Totally agree	1001	84.0	2393	76.8
Professional engagement	Totally disagree	1	0.1	28	1.1
	Partially disagree	13	1.2	51	2.0
	Nor agree, nor disagree	18	1.6	64	2.6
	Partially agree	172	15.5	436	17.3
	Totally agree	906	81.6	1936	77.0

**Table 4:** Comparison of the distribution of the frequency of responses of each questionnaire subsection between principals and teachers.

Subsections		Principals		Teachers		*P*-value
		n	%	n	%	
Teaching planning and practice	Totally disagree	12	0.0	0	0.8	*P*< 0.001
	Partially disagree	23	1.9	4	1.5	
	Nor agree, nor disagree	67	1.4	3	4.3	
	Partially agree	471	18.7	39	30.3	
	Totally agree	982	78.0	163	63.2	
Efficient use of time and material resources during classes	Totally disagree	11	0.0	0	1.0	*P* = 0.008
	Partially disagree	45	7.8	12	4.0	
	Nor agree, nor disagree	81	1.3	2	7.1	
	Partially agree	388	32.5	50	34.1	
	Totally agree	613	58.4	90	53.9	
Strategies and activities to promote/stimulate meaningful learning	Totally disagree	7	0.0	0	0.3	*P* = 0.403
	Partially disagree	24	1.5	5	1.0	
	Nor agree, nor disagree	38	1.8	6	1.6	
	Partially agree	526	24.8	81	21.5	
	Totally agree	1855	71.8	234	75.7	
Knowledge	Totally disagree	1	1.3	1	0.2	*P* = 0.560
	Partially disagree	18	2.6	2	3.2	
	Nor agree, nor disagree	22	3.9	3	3.9	
	Partially agree	171	32.5	25	30.3	
	Totally agree	352	59.7	46	62.4	
Strategies/actions for student assessment	Totally disagree	8	0.5	1	0.5	*P* = 0.326
	Partially disagree	71	2.9	6	4.6	
	Nor agree, nor disagree	55	2.4	5	3.5	
	Partially agree	460	25.4	53	29.7	
	Totally agree	957	68.9	144	61.7	
Comprehensive use of assessment results	Totally disagree	4	0.0	0	0.6	*P* = 0.192
	Partially disagree	6	2.3	2	0.9	
	Nor agree, nor disagree	19	0.0	0	2.9	
	Partially agree	123	13.6	12	18.8	
	Totally agree	502	84.1	74	76.8	
Class atmosphere	Totally disagree	3	0.0	0	0.3	*P* = 0.315
	Partially disagree	13	2.1	3	1.2	
	Nor agree, nor disagree	13	0.0	0	1.2	
	Partially agree	145	9.8	14	13.8	
	Totally agree	875	88.1	126	83.4	
Professional engagement	Totally disagree	1	0.0	0	0.1	*P* = 0.036
	Partially disagree	13	0.0	0	1.3	
	Nor agree, nor disagree	17	0.8	1	1.7	
	Partially agree	160	9.1	12	16.4	
	Totally agree	787	90.2	119	80.5	

## Discussion

This is the first study to examine the characteristics of good teaching based on internationally recognized teaching standards from the perspective of teachers, principals, and students in a Brazilian context. The main results reported here are that the frequencies of responses for the following domains of teaching qualities: “teaching planning and practice”, “efficient use of time and material resources during classes”, and “professional engagement” presented significant differences between the compared groups (i.e., teachers versus principals). For all remaining subsections, we did not find any significant differences between groups.

On average, class atmosphere was the subsection with the highest number of “totally agree” for all assessed groups (Table 1). It is worth noting that four other subsections (i.e., “professional engagement”, “comprehensive use of assessment results”, “strategies and actions for student assessment”, and “teaching planning and practice”) also presented a percentage of “totally agree” responses superior to 70%, which, to some extent, highlights the importance of these teaching qualities for a “good teacher”. Accordingly, within the Brazilian context, previous studies have already detected that the quality of class atmosphere along with a more structured content may positively influence the learning process [30]. Additionally, in other educational systems, these aspects seem to be especially relevant in the practice of good teachers [31, 32]. In fact, it seems that “more skilled teachers” are able to create classes with a good atmosphere and foster school environments that optimize student learning [32]. Thus, based on these observations and according to our results, it is possible to state that, at least for Brazilian principals, teachers, and students, a productive and effective educational system can only be developed by considering not only the training practices and strategies, but also, the whole learning environment [33]. Researchers and educational policy makers are encouraged to consider this multifaceted framework of factors in order to develop more efficient methodological approaches.

As aforementioned, we did not statistically compare students with principals and teachers. However, based on descriptive statistics we decided to discuss differences greater than 10% (Table 2). Under this scenario, students considered the set of qualities “teaching planning and practice” more relevant than principals and teachers (i.e., 64.9% vs 77.1%, for principals/teachers and students, respectively). This emphasizes the importance of teachers carefully planning their classes to promote an efficient and effective learning process, according to the students’ expectations and aims. Reinforcing this rationale, a previous investigation performed in a Brazilian state (i.e., São Paulo) compared the opinions of students regarding the relevance of different work categories for the general progress of society, revealing that, for these young participants, teachers are as important as, for example, doctors and judges [34]. This is counter intuitive and somewhat unexpected in view of the loss of status that the educational segments and occupations have commonly been experiencing in Latin American countries in recent years [35]. “Strategies and actions for student assessment” seem to be even more important under this perspective, as revealed by the greater and robust differences reported here (i.e., 62.6 versus 82.5%, for principals/teachers and students, respectively). In Brazil, this is a critical point, as previous data show that 65% of Brazilian teachers [36] do not feel prepared to deal with their professional challenges on a day-to-day basis, including evaluating students coming from lower socioeconomic backgrounds [37]. It is worth noting that “strategies and actions for student assessment” are usually considered core issues in multiple educational environments, as these evaluations may be implemented to measure learning outcomes and, thus, to guarantee learning equity [38]. Therefore, teaching training programs may incorporate this comprehensive rationality in order to meet the students’ requests and provide adequate and effective teacher development.

Notably, three teaching characteristics presented significant differences between teachers and principals (i.e., “teaching planning and practice”, “efficient use of time and material resources during classes”, and “professional engagement”; Table 3). Concerning “efficient use of time and material resources during classes” teachers agreed that “a good teacher” uses class time according to previous planning, mainly for activities related to teaching and learning, and uses the educational resources available to develop better learning activities, among other peculiarities [39]. Nonetheless, within the Brazilian context, a previous study showed that only 16% of teachers believed that the local government was genuinely engaged with learning processes, contrasting with 84% of teachers who complained about excessive and unnecessary bureaucracy within jeopardy learning environments, which in turn compromise their educational practices [40]. Furthermore, teachers recognized that “good teachers” use school spaces to intensify learning in different areas and spaces, specifically for contextualizing content. Nevertheless, as a baseline for future research, qualitative studies revealed that public schools in Brazil are commonly associated with isolated and segregated places, such as jails or mental hospitals, which probably affects knowledge retention, and learning outcomes [41]. In fact, according to previous analyses, school facilities (e.g., building, classroom, pedagogical equipment) may directly influence comprehension and learning processes [42]. In this sense, the “efficient use of time and material resources during classes” is not only a personal quality for a “good teacher” but also for a “good and efficient educational system”.

With respect to the subsection “professional engagement”, the data show that teachers tend to consider this aspect more important than principals. This result might be driven by the commitment to their own professional development, an understanding that it is important to improve their practice, and the perception of their role to guarantee a good environment in school. This attribute can improve collective work with colleagues [43], leading to a collaborative learning environment, pointing toward potential benefits for both students, teachers, and principals. Specifically in Brazil, a comprehensive examination of school teachers confirmed that 80% of them were willing to collaborate with their peers; however, when asked if they were regularly involved in activities related to peer collaboration, only 33.5% reported maintaining these habits in their daily routines [44]. As such, “professional engagement” should be viewed and managed as a cooperative behavior, able to positively influence the collective workforce of teachers, as well as their development over the years (especially the professional development of novice teachers) [45].

Among these significant differences, surprisingly, “teaching planning and practice” was more important for teachers than principals. In our opinion, this is a point of attention, in terms of time allocation for principals. For example, the Shanghai TALIS 2015 results [46] indicate that for this PISA top performer– 33% of the principals‘ time is dedicated to course- or teaching-related work, while the OCDE average is 21.8%. Contrarily, principals in Shanghai spend less time (34.9%) on administrative affairs than the international average (41.5%). According to the OCDE, this demonstrates that, to some extent, the principals in Shanghai are also teaching experts, rather than administrators who just focus on administration and management. For Brazil (according to TALIS 2013) this is 21% lower than the OCDE average (21.8%) [47]. This could be explained by the fact that in Brazil principals are required to take care of the infrastructure as well as the logistics matters. In Shanghai, these matters are taken care of by private suppliers in some of the top performing schools, which allows principals to spend more time on student-focused activities.

This study is limited by several factors. Firstly, we assessed only students, principals, and teachers who studied and worked in high performance public schools situated in the most developed state of the country (Brazil). Therefore, we cannot extrapolate these data to other environments with inferior levels of excellence. In addition, as this is a cross-sectional study, we cannot establish causal relationships between the examined variables. Nevertheless, regarding what it means to be a “good teacher” in the Brazilian context, the data show that among all groups, the eight dimensions analyzed are important. Overall, all participants agreed or strongly agreed with the importance of each dimension, and also agreed that none of them are considered “unimportant”. This is a relevant finding because it appears that all groups agree upon what constitutes good teaching within the actual Brazilian context. To properly develop these set of qualities and skills, it seems that traditional educational methods are not sufficient. As such, integrated solutions and strategies may be recommended in conjunction with more multidimensional teaching approaches such as, for example, integral teaching and student development [48–50]. Further studies should be conducted to examine the effects of these multifaceted strategies on the professional development of “good teachers”.

## Conclusions

This study has important implications for teaching and learning evolution within the Brazilian educational context. In conclusion, we demonstrated that some teaching characteristics, such as “class atmosphere”, “professional engagement”, “efficient use of assessment results”, “strategies and actions for student assessment”, and “teaching planning and practice” are more important than others, with some qualities and competencies exhibiting significant differences and distinct levels of importance between groups (i.e., “teaching planning and practice”, “efficient use of time and material resources during classes”, and “professional engagement”, when comparing teachers and principals). However, it is crucial to emphasize that all assessed educational domains may be recognized as critical and essential teaching characteristics, as all of them presented high levels of “totally agree” responses among the three assessed groups (i.e., ≥ 57.9%, on average, for principals, teachers, and students). These novel and comprehensive findings may help government agencies and researchers to develop better and more tailored policies and teacher training programs, specifically designed for preparing middle-school teachers as effective educators and transformational leaders in the Brazilian scenario. Given the importance and relevance of education as a means of social mobility and economic development, we firmly believe that this type of information can not only move the field forward, but also impact the school environment and, in a broader context, the development of the country.

## Supporting information

S1 File (DOCX)

S2 File (DOCX)
