# Supplementary material for: What teachers need to know and be able to do: A view from teachers, students, and principals in the Brazilian context
Source: PLoS One. 2020 Sep 14;15(9):e0238990. doi: 10.1371/journal.pone.0238990 (PMC7489549; doi:10.1371/journal.pone.0238990)
Supplement: S1 File — (DOCX) [file pone.0238990.s001.docx]

**
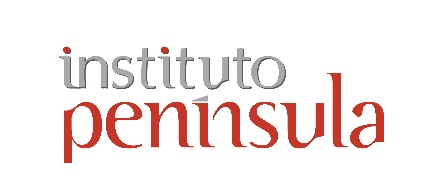

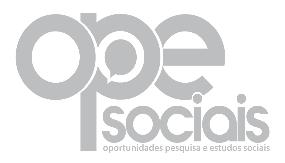
**

**PESQUISA BOM PROFESSOR**

**Questionário do Estudante**

| **ESCOLA:** NOME + INEP (INFORMAÇÕES VARIAVÉIS SERÁ ENVIADA PARA EMPRESA DE LEITURA ÓPTICA INSERIR | |
| --- | --- |
| **CÓDIGO DE IDENTIFICAÇÃO**: INFORMAÇÕES VARIÁVEIS A PARTIR DO CÓDIGO GERADO PELA OPE SOCAIS QUE SERÁ ENVIADA PARA EMPRESA DE LEITURA ÓPTICA INSERIR  **TURMA:** (INFORMAÇÕES VARIÁVEIS) SERÁ ENVIADA PARA EMPRESA DE LEITURA ÓPTICA INSERIR  **TURNO:** (INFORMAÇÕES VARIÁVEIS) SERÁ ENVIADA PARA EMPRESA DE LEITURA ÓPTICA INSERIR | |
| **Olá Estudante, tudo bom?**  Você está sendo convidado a participar, junto ao Instituto Península, da pesquisa de opinião “Bom Professor”, sobre as boas práticas da região, que tem avançado tanto na valorização da carreira do seu professor, como no entendimento do que é ser um bom professor pela sua comunidade.  A sua participação é muito importante, pois nos ajuda a construir e a alimentar essas reflexões, além de ajudar na formulação da BNCFD - Base Nacional Comum de Formação de Docente, que atualmente está sendo debatida e construída pelo Conselho Nacional de Educação, com o objetivo de melhorar o ensino que é oferecido aos estudantes, assim como você.  **Pode ficar tranquilo, não há respostas certas ou erradas neste questionário, só queremos escutar sua voz e suas percepções sobre alguns assuntos. Beleza? Vamos lá!** | |
| **IDENTIFICAÇÃO** | 1. **COR OU RAÇA (opcional):**   ⬜ PRETA  ⬜ PARDA  ⬜ BRANCA  ⬜ AMARELA (ORIGEM ASIÁTICA)  ⬜ INDÍGENA |
| 1. **DATA DE NASCIMENTO: CAMPO ABERTO PARA O ALUNO ESCREVER A EMPRESA DE LEITURA ÓPTICA VAI POR QUADRADINHOS NESTA PARTE** |  |
| 1. **SEXO (opcional): ⬜** FEMININO ⬜ MASCULINO |  |
| As respostas das perguntas vão de 1 a 5, sendo:  **1 = Discordo totalmente**  **2 = Discordo parcialmente**  **3 = Nem concordo, nem discordo**  **4 = Concordo parcialmente**  **5 = Concordo totalmente**  Assinale abaixo, a opção que mais se encaixa a partir **das suas percepções**. |  |

| PARTE 1 - Para mim, um bom professor ou uma boa professora: | 1 | 2 | 3 | 4 | 5 |
| --- | --- | --- | --- | --- | --- |
| 1. Organiza bem as atividades. |  |  |  |  |  |
| 1. Fala com clareza para que possamos entender os conteúdos. |  |  |  |  |  |
| 1. Mostra clareza ao longo do ano. |  |  |  |  |  |
| 1. Chega para dar aula com sua aula planejada. |  |  |  |  |  |
| PARTE 2 - Para mim, um bom professor ou uma boa professora precisa: | 1 | 2 | 3 | 4 | 5 |
| 1. Nos dar tempo durante as aulas para explicar nossas ideias. |  |  |  |  |  |
| 1. Demonstrar tranquilidade quanto ao tempo necessário para que possamos fazer as atividades. |  |  |  |  |  |
| 1. Evitar atrasos excessivos de conteúdo. |  |  |  |  |  |
| PARTE 3 - Para mim, um bom professor ou uma boa professora precisa: | 1 | 2 | 3 | 4 | 5 |
| 1. Entender qual parte da matéria temos mais dificuldade. |  |  |  |  |  |
| 1. Perceber quando estamos ficando entediados com a aula. |  |  |  |  |  |
| 1. Ter ou Demonstrar interesse quando estivermos explicando nossas respostas ou quando estivermos falando sobre nossos pensamentos. |  |  |  |  |  |
| 1. Relacionar os exemplos da matéria com nossa realidade (casa, amigos, redes sociais, recreio, etc). |  |  |  |  |  |
| PARTE 4 - Para mim, na prática, um bom professor ou uma boa professora precisa: | 1 | 2 | 3 | 4 | 5 |
| 1. Saber do que está falando. |  |  |  |  |  |
| 1. Ter domínio do conteúdo, mesmo que não consiga ensiná-lo. |  |  |  |  |  |
| 1. Ser amável e próximo dos alunos, mesmo que não domine o conteúdo. |  |  |  |  |  |
| 1. Aceitar que as vezes não vai conhecer todo o conteúdo e quando isso acontecer, precisa se comprometer a trazer essa informação na próxima aula. |  |  |  |  |  |
| PARTE 5 - Para mim, um bom professor ou uma boa professora precisa: | 1 | 2 | 3 | 4 | 5 |
| 1. Não só esperar que memorizemos, mas também que pensemos sobre a matéria. |  |  |  |  |  |
| 1. Me ajudar a refletir e entender por que errei. |  |  |  |  |  |
| 1. Usar diferentes métodos de avaliação juntos (nota da prova, participação em sala, produção individual, trabalhos em grupo etc.) |  |  |  |  |  |
| PARTE 6 - Para mim, um bom professor ou uma boa professora: | 1 | 2 | 3 | 4 | 5 |
| 1. Me anima a dar o meu melhor nas atividades. |  |  |  |  |  |
| 1. Me incentiva a melhorar mesmo após um resultado abaixo do esperado. |  |  |  |  |  |
| 1. Me motiva a apoiar os colegas que não se deram bem nas provas. |  |  |  |  |  |
| 1. Me incentiva a buscar apoio quando preciso e a dar apoio quando alguém pede. |  |  |  |  |  |
| PARTE 7 - Para mim, um bom professor ou uma boa professora precisa: | 1 | 2 | 3 | 4 | 5 |
| 1. Nos ajudar a refletir para tratar a todos com cuidado e respeito. |  |  |  |  |  |
| 1. Inspirar esforço e dedicação entre nós. |  |  |  |  |  |
| 1. Dar liberdade para que façamos perguntas e demos nossa opinião. |  |  |  |  |  |
| 1. Fazer com que nos sintamos inteligentes. |  |  |  |  |  |
| 1. Respeitar nossas ideias e sugestões. |  |  |  |  |  |
| PARTE 8 - Para mim, um bom professor ou boa professora precisa: | 1 | 2 | 3 | 4 | 5 |
| 1. Se preocupar com a minha aprendizagem. |  |  |  |  |  |
| 1. Nos ensinar sobre o valor da educação em nossa vida. |  |  |  |  |  |
| 1. Demonstrar gosto pela profissão. |  |  |  |  |  |
| 1. Sempre fazer coisas legais para ensinar sem ser chato. |  |  |  |  |  |
