# Supplementary material for: What teachers need to know and be able to do: A view from teachers, students, and principals in the Brazilian context
Source: PLoS One. 2020 Sep 14;15(9):e0238990. doi: 10.1371/journal.pone.0238990 (PMC7489549; doi:10.1371/journal.pone.0238990)
Supplement: S2 File — (DOCX) [file pone.0238990.s002.docx]

**
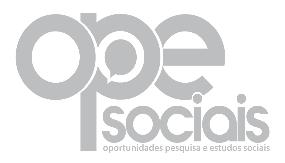

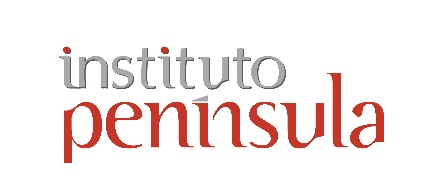
PESQUISA BOM PROFESSOR**

**Questionário para Gestores e Professores**

| **ESCOLA:** NOME + INEP (INFORMAÇÕES VARIAVÉIS)  **CÓDIGO DE IDENTIFICAÇÃO**: INFORMAÇÕES VARIÁVEIS A PARTIR DO CÓDIGO GERADO PELA OPE SOCAIS QUE SERÁ ENVIADA PARA EMPRESA DE LEITURA ÓPTICA INSERIR | |
| --- | --- |
| **Olá Gestor(a) e Professor(a), tudo bom?**  Você está sendo convidado a participar da pesquisa de opinião “Bom Professor”, nós do Instituto Península estamos pesquisando sobre as boas práticas da região que tem avançado tanto na valorização da carreira do docente, como na percepção (e autopercepção) do que significa ser um bom professor. Gostaríamos de construir e nutrir essas reflexões junto com vocês!  A sua participação é muito importante, pois esta é uma pesquisa de opinião com o objetivo de dar insumos quantitativos na discussão da formulação da BNCFD - Base Nacional Comum de Formação de Docentes que atualmente está sendo debatida e construída pelo Conselho Nacional de Educação. Não há respostas certas ou erradas neste questionário, só queremos dar voz tanto a professores quanto aos alunos para alimentar a discussão com dados reais sobre a percepção do que significa ser um bom professor nas 7 melhores escolas (fund 2) segundo IDEB . | |
|  | |
| **IDENTIFICAÇÃO** | |
|  | 1. **DISCIPLINA QUE LECIONA:**   **⬜** MATEMÁTICA  ⬜ HISTÓRIA  ⬜ GEOGRAFIA  ⬜ LÍNGUA PORTUGUESA  ⬜ CIÊNCIAS  ⬜ LÍNGUA ESTRANGEIRA  ⬜ OUTRO (ESPECIFICAR): |
|  |  |
| 1. **SEXO (opcional): ⬜** FEMININO ⬜ MASCULINO |  |
| 1. **FUNÇÃO:**   **⬜** DIRETOR  ⬜ COORDENADOR PEDAGÓGICO  ⬜ PROFESSOR(A) |  |

As respostas das perguntas vão de 1 a 5, sendo:

**1 = Discordo totalmente**

**2 = Discordo parcialmente**

**3 = Nem concordo, nem discordo**

**4 = Concordo parcialmente**

**5 = Concordo totalmente**

Assinale a opção que mais se encaixa com suas percepções.

| **PARTE 1 - PLANEJAMENTO DA PRÁTICA DOCENTE** | | | | | |
| --- | --- | --- | --- | --- | --- |
| Como profissional, um bom professor ou uma boa professora precisa: | 1 | 2 | 3 | 4 | 5 |
| 1. Dedicar de maneira periódica um tempo específico para planejar o trabalho docente |  |  |  |  |  |
| 2. Realizar um planejamento escrito das aulas |  |  |  |  |  |
| 3. Entender contextos refletindo sobre a perspectiva dos alunos e pares para incluir diversas perspectivas |  |  |  |  |  |
| No planejamento das aulas, um bom professor ou uma boa professora precisa: | 1 | 2 | 3 | 4 | 5 |
| 4. Consultar o Projeto Político Pedagógico (PPP) da escola para ter presentes os princípios gerais em todos os componentes curriculares. |  |  |  |  |  |
| 5. Consultar o Projeto Político Pedagógico (PPP) para considerar a sequência, profundidade e inter-relação entre os diversos componentes de ensino. |  |  |  |  |  |

As respostas das perguntas vão de 1 a 5, sendo:

**1 = Discordo totalmente**

**2 = Discordo parcialmente**

**3 = Nem concordo, nem discordo**

**4 = Concordo parcialmente**

**5 = Concordo totalmente**

Assinale a opção que mais se encaixa com suas percepções.

|  | 1 | 2 | 3 | 4 | 5 |
| --- | --- | --- | --- | --- | --- |
| 6. Consultar as diretrizes da Secretaria de Educação para identificar as recomendações didáticas de ensino, a forma como devem ser empregadas e sugestões para realizar as avaliações dos alunos. |  |  |  |  |  |
| 7. Consultar as diretrizes da Secretaria de Educação para identificar quais atividades devem ser realizadas de cada componente curricular. |  |  |  |  |  |
| 8. Consultar materiais referentes às didáticas relacionadas às áreas do conhecimento. |  |  |  |  |  |
| 9. Consultar materiais de formação e atualização para retomar informações sobre os conteúdos conceituais, procedimentais e atitudinais e as formas de ensiná-los. |  |  |  |  |  |
| 10. Consultar os materiais didáticos utilizados por exemplo: Olimpíada da Língua Portuguesa e Matemática, assim como as edições do Prêmio Professores do Brasil. |  |  |  |  |  |
| 11. Consultar as histórias e realidades de cada turma para refletir sobre o melhor planejamento que estimule o aprendizado dos alunos. |  |  |  |  |  |
|  |  |  |  |  |  |
| No plano/diário de aula um bom professor ou uma boa professora precisa estabelecer: | 1 | 2 | 3 | 4 | 5 |
| 12. Os conteúdos de ensino considerando os componentes ou eixos temáticos de cada área. |  |  |  |  |  |
| 13. Os objetivos de aprendizagem que precisam ser atingidos com os alunos assim como o desenvolvimento de habilidades, atitudes e aquisição de conhecimentos. |  |  |  |  |  |
| 14. A forma como serão ensinados os objetos de conhecimento, estabelecendo os tipos de atividades e formas de participação tanto dos alunos quanto do professor. |  |  |  |  |  |
| 15. A ordem e os momentos nos quais as atividades serão implementadas. |  |  |  |  |  |
| 16. Os recursos que vão ser empregados para implementar as atividades de ensino. |  |  |  |  |  |
| 17. O que será avaliado nos alunos: conhecimentos (conceitos), habilidades (procedimentos) e atitudes. |  |  |  |  |  |
| 18. As estratégias de avaliação para avaliar o aprendizado dos alunos. |  |  |  |  |  |
| 19. Momentos nos quais serão desenvolvidas estratégias de avaliação (elaborar registros, aplicar provas, fazer observações, etc). |  |  |  |  |  |
|  |  |  |  |  |  |
| **PARTE 2 - APROVEITAMENTO DO TEMPO E RECURSOS MATERIAIS PARA DESENVOLVER AS AULAS** | | | | | |
| Ao desenvolver as aulas um bom professor ou uma boa professora utiliza: | 1 | 2 | 3 | 4 | 5 |
| 20. O tempo das atividades de acordo ao que já tinha sido estimado ou planejado. |  |  |  |  |  |
| 21. Pouco tempo de transição entre uma atividade e outra. |  |  |  |  |  |
| 22. A maior parte do tempo em atividades relacionadas com ensino e aprendizagem. |  |  |  |  |  |
| 23. Recursos da Secretaria de Educação para fomento ao desenvolvimento de atividade de ensino e avaliação e também para identificar a forma e o momento a serem utilizados. |  |  |  |  |  |
| 24. Estratégias variadas para intensificar a aprendizagem. |  |  |  |  |  |
| 25. Materiais diversos para desenvolver habilidades e atitudes sobre hábito de leitura. |  |  |  |  |  |
| 26. Imagens informativas que contribuem para a construção de conhecimentos. |  |  |  |  |  |
| 27. Jogos didáticos como estratégia de mobilização de conhecimentos e intensificação dos aprendizados dentro da sala de aula. |  |  |  |  |  |
| 28. Materiais reutilizáveis e de papelaria para apoiar aprendizagem de noções, conceitos e para promover o desenvolvimento das habilidades de expressão. |  |  |  |  |  |
| 29. A lousa para troca de informações entre alunos e professor, para que registrem informações importantes e realizem exercícios em conjunto, além de ser fonte de consulta e monitoramento. |  |  |  |  |  |
| 30. Cadernos dos alunos e anotações usados para registrar informação e também como fonte de consulta e monitoramento. |  |  |  |  |  |
| 31. Espaços da escola (quadra, outras salas, exteriores, etc) para intensificar o ensino das diversas temáticas. |  |  |  |  |  |
| 32. Recursos da natureza para contextualizar as temáticas. |  |  |  |  |  |
| 33 Lugares do bairro e município para enriquecer o ensino de diversas temáticas (museus, jardins, estradas, fábricas, campos, etc) |  |  |  |  |  |
| As respostas das perguntas vão de 1 a 5, sendo:  **1 = Discordo totalmente**  **2 = Discordo parcialmente**  **3 = Nem concordo, nem discordo**  **4 = Concordo parcialmente**  **5 = Concordo totalmente**  Assinale a opção que mais se encaixa com suas percepções.  **PARTE 3 - ESTRATÉGIAS E ATIVIDADES PARA PROMOVER APRENDIZAGENS SIGNIFICATIVAS** | | | | | |
| Ao desenvolver as aulas um bom professor ou uma boa professora precisa: | 1 | 2 | 3 | 4 | 5 |
| 34. Dar instruções completas para que os alunos realizem as atividades (sinaliza o que será realizado, indica os materiais e recursos, a forma de se organizar, o tempo para fazer a atividade, etc) |  |  |  |  |  |
| 35. Verificar se as instruções foram compreendidas para os alunos. |  |  |  |  |  |
| 36. Promover atividades em grupo, dinamizando o trabalho entre os alunos. |  |  |  |  |  |
| 37. Organizar os alunos para que realizem atividades em duplas. |  |  |  |  |  |
| 38. Atribuir atividades para que os alunos as façam de maneira independente. |  |  |  |  |  |
| 39. Atribuir aos alunos com maior fluidez no desempenho, diferentes funções para que participem apoiando seus colegas assim como no desenvolvimento da aula. |  |  |  |  |  |
| 40. Promover o trabalho colaborativo entre os alunos para que interajam e se ajudem. |  |  |  |  |  |
| 41. Apoiar individualmente os alunos que não consigam realizar atividades por conta própria. |  |  |  |  |  |
| 42. Dedicar cuidado especial aos alunos com maior defasagem, por meio da revisão pontual dos seus trabalhos, atribuição das atividades e promoção permanente da participação. |  |  |  |  |  |
| 43. Propor situações de leitura que mobilizem os alunos e que, com isso, participem de distintas formas: fazendo predições, antecipações, comentando as leituras, etc |  |  |  |  |  |
| 44. Fomentar a realização de demonstrações para explicar passo a passo os processos para elaboração de trabalhos e exercícios. |  |  |  |  |  |
| 45. Promover que os alunos produzam textos variados com diferentes funções: adquirir uma informação de caráter geral ou específica, estudar, realizar uma atividade prática, escrever, comunicar algo em voz alta e praticar leitura em voz alta, etc. |  |  |  |  |  |
| 46. Promover atividades nas quais os alunos obtenham informação por meio de diferentes fontes (pessoas da escola, do bairro, materiais impressos, vídeos, etc) |  |  |  |  |  |
| 47. Promover que os alunos elaborem conclusões e inferências a partir das análises de informação. |  |  |  |  |  |
| 48. Promover que os alunos realizem atividades com as quais consigam desenvolver habilidades de representação, tais como elaboração de modelos, mapas, gráficos, etc. |  |  |  |  |  |
| 49. Realizar atividades para que os alunos aprendam a organizar e apresentar informação por meio de diversas formas (esquemas, figuras, quadros, textos, etc) |  |  |  |  |  |
| 50. Promover que os alunos leiam diferentes tipos de textos (contos, crônicas, notícias, artigos de opinião, instruções de uso; ensaios; relatos de experiência vivida; etc) |  |  |  |  |  |
| 51. Realizar atividades com o objetivo de desenvolver habilidades de pesquisa nos alunos, tais como observação, entrevista, etc. |  |  |  |  |  |
| 52. Promover atividades com o objetivo de que os alunos implementem, analisem, sintetizem e avaliem o aprendido |  |  |  |  |  |
| 53. Apresentar os objetos de conhecimento e temáticas a partir de situações familiares para os alunos e implementar em situações reais. |  |  |  |  |  |
| 54. Promover situações que os alunos apliquem os aprendizados em contextos ou situações novas. |  |  |  |  |  |
| 55. Promover a participação da maioria dos alunos nas atividades grupais e apresentações. |  |  |  |  |  |
| 56. Promover que os alunos participem das propostas de atividades por iniciativa própria. |  |  |  |  |  |
| 57. Promover que os alunos expliquem sobre a forma como realizam seus trabalhos e os resultados obtidos a partir disso. |  |  |  |  |  |
| 58. Promover que os alunos expressem suas ideias e argumentem suas respostas. |  |  |  |  |  |
| 59. Realizar atividades nas quais os alunos narrem acontecimentos, experiências, etc. |  |  |  |  |  |
| 60. Promover que os alunos compartilhem e comparem suas ideias e opiniões. |  |  |  |  |  |
| 61. Promover que os alunos expressem os aprendizados com suas próprias palavras. |  |  |  |  |  |
| 62. Realizar atividades variadas para tratar de atender características e necessidades da turma, incluindo: alunos com defasagem, desempenho médio ou com desempenho superior e com necessidades educacionais especiais. |  |  |  |  |  |
| 63. Realizar atividades de maneira que a maioria dos alunos possam desempenhá-las com sucesso e dentro do tempo estimado. |  |  |  |  |  |

As respostas das perguntas vão de 1 a 5, sendo:

**1 = Discordo totalmente**

**2 = Discordo parcialmente**

**3 = Nem concordo, nem discordo**

**4 = Concordo parcialmente**

**5 = Concordo totalmente**

Assinale a opção que mais se encaixa com suas percepções.

| **PARTE 4 - CONHECIMENTO** | | | | | |
| --- | --- | --- | --- | --- | --- |
| Na prática, um bom professor ou uma boa professora precisa: | 1 | 2 | 3 | 4 | 5 |
| 64. Dominar os conteúdos (conceitos, procedimentos e atitudes) e saber como ensiná-los |  |  |  |  |  |
| 65. Dominar os conceitos, princípios e estruturas do conteúdo da área. |  |  |  |  |  |
| 66. Dominar as competências gerais e específicas da área e os objetos de conhecimento da docência estabelecidos na Base Nacional Comum Curricular e expressos no currículo da escola |  |  |  |  |  |
| 67. Conhecer a estrutura e a governança dos sistemas educacionais |  |  |  |  |  |
| 68. Evocar as questões filosóficas e históricas a respeito da constituição da escola e das práticas educacionais |  |  |  |  |  |
| 69. Interpretar a estrutura do sistema educacional brasileiro, as formas de gestão, as políticas e programas e a legislação vigentes. |  |  |  |  |  |
| 70. Examinar, analisar, criar estratégias a partir dos resultados de avaliações em larga escala. |  |  |  |  |  |
| **PARTE 5 - ESTRATÉGIAS E AÇÕES PARA AVALIAÇÃO DOS ALUNOS** | | | | | |
| Para avaliar os alunos, um bom professor ou uma boa professora precisa: | 1 | 2 | 3 | 4 | 5 |
| 71. Realizar diagnóstico ao início do ciclo escolar, para medir os conhecimentos prévios dos alunos. |  |  |  |  |  |
| 72. Realizar atividades variadas para conhecer nível de conhecimento dos alunos sobre cada temática (jogos, interrogatório, resolução de problemas, etc) |  |  |  |  |  |
| 73. Empregar estratégias de avaliação escrita: exames, questionários, etc. |  |  |  |  |  |
| 74. Garantir que exames escritos aplicados tenham elementos e características apropriadas. |  |  |  |  |  |
| 75. Empregar estratégias de avaliação oral: entrevistas, discussão oral, etc. |  |  |  |  |  |
| 76. Elaborar permanentemente registros que ajudem a identificar avanços e progressos dos alunos (registros de observação, entrevista, etc) |  |  |  |  |  |
| 77. Organizar registros de informação como resultados das provas, valorações dos trabalhos, participação dos alunos, cumprimento dos compromissos, etc |  |  |  |  |  |
| 78. Organizar um registro por turma, assim como um portfólio de avanços, ferramentas que dão conta dos seus progressos. |  |  |  |  |  |
| 79. Verificar que as atividades propostas sejam realizadas sem dificuldades. |  |  |  |  |  |
| 80. Revisar com agilidade os trabalhos e lições de casa, para identificar ganhos e dificuldades. |  |  |  |  |  |
| 81. Revisar os exercícios dos cadernos e livros. |  |  |  |  |  |
| 82. Promover atividades de autoavaliação. |  |  |  |  |  |
| 83. Promover atividades de co-avaliação (entre alunos). |  |  |  |  |  |
| 84. Realizar avaliações periódicas. |  |  |  |  |  |
| Para qualificar os alunos, um bom professor ou uma boa professora precisa: | 1 | 2 | 3 | 4 | 5 |
| 85. Utilizar os resultados das provas e exercícios escritos. |  |  |  |  |  |
| 86. Considerar resultados das provas orais. |  |  |  |  |  |
| 87. Levar em conta a informação dos registros dos trabalhos escritos. |  |  |  |  |  |
| 88. Considerar informação da participação dos alunos nas atividades. |  |  |  |  |  |
| 89. Considerar os resultados dos registros de cumprimento de lições de casa ou tarefas. |  |  |  |  |  |
| As respostas das perguntas vão de 1 a 5, sendo:  **1 = Discordo totalmente**  **2 = Discordo parcialmente**  **3 = Nem concordo, nem discordo**  **4 = Concordo parcialmente**  **5 = Concordo totalmente**  Assinale a opção que mais se encaixa com suas percepções. |  |  |  |  |  |
| **PARTE 6 - USO DOS RESULTADOS DE AVALIAÇÃO** | | | | | |
| Um bom professor ou uma boa professora utiliza as avaliações para: | 1 | 2 | 3 | 4 | 5 |
| 90. Identificar necessidades de apoio para os alunos. |  |  |  |  |  |
| 91. Decidir ações de apoio conjuntamente com as famílias. |  |  |  |  |  |
| 92. Ajustar o trabalho docente no planejamento das aulas. |  |  |  |  |  |
| 93. Ajustar o trabalho docente nos materiais e recursos educativos a serem utilizados. |  |  |  |  |  |
| 94. Ajustar a prática docente nas estratégias de trabalho usadas. |  |  |  |  |  |
| 95. Ajustar o trabalho docente nos instrumentos e critérios de avaliação. |  |  |  |  |  |
| 96. Informar aos alunos os seus avanços, progressos e aspectos que tem para melhorar. |  |  |  |  |  |
| 97. Informar às famílias dos alunos os avanços, progressos e aspectos nos quais são necessários apoiar. |  |  |  |  |  |
| **PARTE 7 - CLIMA DE AULA** | | | | | |
| Na sala de aula, um bom professor ou uma boa professora fomenta: | 1 | 2 | 3 | 4 | 5 |
| 98. Que sejam estabelecidas normas de disciplina com a aprovação de todos |  |  |  |  |  |
| 99. Respeito e aceitação as normas de disciplina acordadas. |  |  |  |  |  |
| 100. Aplicação das normas acordadas para manter a sala de aula ordenada e limpa. |  |  |  |  |  |
| 101. Aplicação das normas para criar um clima de aula que favoreça o aprendizado. |  |  |  |  |  |
| Em relação ao clima da aula, um bom professor ou uma boa professora deve: | 1 | 2 | 3 | 4 | 5 |
| 102. Fomentar nos alunos a expressão livre das ideias e sentimentos sem medo de serem ridicularizados ou sancionados. |  |  |  |  |  |
| 103. Se preocupar por conhecer interesses, gostos, tendências e necessidades dos alunos conversando com eles, seus pais e familiares. |  |  |  |  |  |
| 104. Estar atento às relações entre os alunos. |  |  |  |  |  |
| 105. Estabelecer um trato igual sem instaurar favoritismos. |  |  |  |  |  |
| 106. Manifestar preocupação e afeto pelos alunos por meio de diferentes formas (conversando, enviando material extra, etc). |  |  |  |  |  |
| 107. Motivar os alunos para engajá-los a participarem de todas as atividades. |  |  |  |  |  |
| 108. Promover no momento oportuno a resolução dos conflitos. |  |  |  |  |  |
| 109. Manter a calma ante situações conflitivas evitando reações negativas como gritar, golpear objetos, etc. |  |  |  |  |  |
| 110. Promover um ambiente de coleguismo e solidariedade entre alunos. |  |  |  |  |  |
| **PARTE 8 - ENGAJAMENTO PROFISSIONAL** | | | | | |
| As responsabilidades profissionais de um bom professor ou de uma boa professora são: | 1 | 2 | 3 | 4 | 5 |
| 111. Comprometer-se com o próprio desenvolvimento profissional. |  |  |  |  |  |
| 112. Assumir a responsabilidade do aprimoramento da prática, participando de atividades formativas e/ou desenvolvendo atividades com colegas. |  |  |  |  |  |
| 113. Engajar-se em estudos ou pesquisas de problemas de educação (nas diversas modalidades) e na busca de soluções. |  |  |  |  |  |
| 114. Demonstrar conhecimento das competências gerais da Base Nacional Comum Curricular. |  |  |  |  |  |
| 115. Trabalhar coletivamente com seus colegas de escola, bairro ou unidade federativa. |  |  |  |  |  |

As respostas das perguntas vão de 1 a 5, sendo:

**1 = Discordo totalmente**

**2 = Discordo parcialmente**

**3 = Nem concordo, nem discordo**

**4 = Concordo parcialmente**

**5 = Concordo totalmente**

Assinale a opção que mais se encaixa com suas percepções.

| As responsabilidades profissionais de um bom professor ou de uma boa professora são: | 1 | 2 | 3 | 4 | 5 |
| --- | --- | --- | --- | --- | --- |
| 116. Conhecer, entender e valorizar as diferentes identidades dos estudantes e ser capaz de utilizar a diversidade como recurso pedagógico. |  |  |  |  |  |
| 117. Contribuir na construção e na avaliação do projeto pedagógico da escola zelando pela prioridade da aprendizagem dos estudantes. |  |  |  |  |  |
| 118. Comunicar e interagir com as famílias para estabelecer parcerias e colaboração com a escola em busca da garantia da aprendizagem dos estudantes. |  |  |  |  |  |
| 119. Compartilhar responsabilidades e construir clima escolar favorável ao desempenho das atividades com os colegas e gestão. |  |  |  |  |  |
| A ética de um bom professor ou de uma boa professora é, entre outros aspectos: | 1 | 2 | 3 | 4 | 5 |
| 120. Acreditar que todos os alunos podem aprender. |  |  |  |  |  |
| 121. Acreditar na importância do aprimoramento dos seus conhecimentos e continuar aprendendo a vida inteira. |  |  |  |  |  |
| 122. Acreditar no poder transformador da educação para garantir uma sociedade com oportunidades para todos. |  |  |  |  |  |
